# Possible protective effect of rosuvastatin in chemotherapy-induced cardiotoxicity in HER2 positive breast cancer patients: a randomized controlled trial

**DOI:** 10.1007/s12032-024-02426-1

**Published:** 2024-07-08

**Authors:** Khlood M. Kettana, Sahar M. El‑Haggar, Mohamed A. Alm El-Din, Dalia R. El‑Afify

**Affiliations:** 1 Clinical Pharmacy Department, Faculty of Pharmacy, Tanta University, Tanta, Egypt; 2 Clinical Oncology Department, Faculty of Medicine, Tanta University, Tanta, Egypt

**Keywords:** Rosuvastatin, Doxorubicin, Trastuzumab, Cardiotoxicity

## Abstract

Cardiotoxicity is a side effect of chemotherapy in human epidermal growth factor receptor 2 (HER2) positive breast cancer patients receiving both anthracyclines and trastuzumab. We looked for a possible protective effect of rosuvastatin against chemotherapy-induced cardiotoxicity. Methods: 50 newly diagnosed HER2 positive breast cancer patients were randomly allocated into two groups: 25patients in each. Group 1(control group) received doxorubicin for 4 cycles (3 months) followed by trastuzumab adjuvant therapy. Group 2 (treatment group) received doxorubicin for 4 cycles (3 months) followed by trastuzumab adjuvant therapy and 20 mg of oral rosuvastatin 24 h before the first cycle of chemotherapy and once daily for the rest of the follow-up period (6 months). Transthoracic echocardiography was done, and blood samples were collected for patients 24 h before the initiation of therapy, after 3 months and after 6 months to assess serum levels of high sensitivity cardiac troponin I (hs-cTnI), Myeloperoxidase (MPO), Interleukin-6 (IL-6) and Alanine aminotransferase (ALT). The study was retrospectively registered in Clinical Trials.gov in April 2022. Its ID is NCT05338723. Compared to control group, Rosuvastatin-treated group had a significantly lower decline in LVEF after 3 months and after 6 months. They had significantly lower Hs-cTnI and IL-6 after 3 months and after 6 months, and significantly lower MPO after 6 months. Four patients in control group experienced cardiotoxicity while no one in rosuvastatin-treated group. Rosuvastatin attenuated cardiotoxicity, so it is a promising protective agent against chemotherapy-induced cardiotoxicity.

## Introduction

Breast cancer accounts for 30% of female cancers [1]. It is the most common cause of cancer-related death in females worldwide [2]. About 15% to 20% of breast cancers are human epidermal growth factor receptor 2(HER2) positive [2]. HER2 positive breast cancer confers an aggressive clinical phenotype including accelerated cell growth, high risk of systemic metastasis and recurrence [3].To improve treatment outcome of this aggressive breast cancer, patients are treated with doxorubicin-based chemotherapy followed by adjuvant trastuzumab which is monoclonal antibody directed against HER2 receptors [4]. Clinical trials showed that administration of trastuzumab sequential with doxorubicin was associated with high levels of cardiotoxicity as it interferes with homeostatic mechanisms and pathways of cell survival and repair, which exacerbates the damage induced by prior doxorubicin therapy [5]. Doxorubicin, as a member of anthracyclines, causes type 1 cardiotoxicity which is non-revesible, dose dependent and represented by structural cardiomyocyte alterations and cell death due to production of reactive oxygen species (ROS) which interacts with the myocardium and leads to imbalance between antioxidant mechanisms and pro-inflammatory substances [5, 6]. While doxorubicin increases the production of reactive oxygen and nitrogen species (ROS/RNS), blocking HER-2 signalling decreases activation of survival pathways and worsens oxidative and nitrative stress. This is the probable explanation for the additive cardiotoxic effect [7]. Trastuzumab causes type 2 cardiotoxicity as it inhibits signal transduction, neoangiogenesis and repair of DNA damage. Its cardiotoxicity is often reversible and doesn’t depend on the dose [5].

Many clinical variables predispose patients to this unfavorable adverse effect. Besides old age, history of cardiac dysfunction, diabetes and hypertension, racial and ethnic differences are known to affect incidence of cardiotoxicity in those patients [8, 9].

In oncological clinical practice, transthoracic echocardiography (ECHO) is the most often used diagnostic method for assessing cardiotoxicity as it is used for periodic evaluation of cardiac function and detects any changes in left ventricular ejection fraction (LVEF) [6].For early prediction of cardiotoxicity, serum levels of high sensitivity cardiac troponin I (hs-cTnI), Myeloperoxidase (MPO) and Interleukin-6 (IL-6) can be assessed [10–12].Multiple strategies are used to prevent chemotherapy-induced cardiotoxicity as reduction of the dose of chemotherapy or the use of cardioprotective drugs [6].Statins, including rosuvastatin, 3-hydroxy-3-methylglutaryl-coenzyme A (HMG-CoA) reductase inhibitors, are widely used cholesterol lowering drugs. Rosuvastatin is believed to have antioxidant and anti-inflammatory effects that may help to prevent DNA damage and provide cardioprotective effect [13].

In this study, we investigate the possible protective effect of rosuvastatin in chemotherapy-induced cardiotoxicity in HER2 positive breast cancer patients.

### Patients and methods

Our study was a prospective, randomized, controlled, parallel study conducted on 50 HER2 positive breast cancer patients that received doxorubicin followed by trastuzumab adjuvant therapy. The study was approved by The Research Ethics Committee of Tanta University and was carried out in compliance with the Declaration of Helsinki.All patients gave their written informed consents before participation. The study was retrospectively registered in Clinical Trials.gov and its ClinicalTrials.gov ID is NCT05338723. We used the CONSORT reporting guidelines [14].

### Inclusion criteria

Female patients aged between 25 and 75 years old who were newly diagnosed with HER2 positive breast cancer and scheduled to receive doxorubicin followed by trastuzumab adjuvant therapy, with Eastern Cooperative Oncology Group (ECOG) performance status ≤ 2, preserved left ventricular (LV) systolic function in which the left ventricular ejection fraction (LVEF) ≥ 50%,normal hematological, renal function and Alanine amino transferase (ALT) ≤ 3 times upper limit of normal (ULN) were included in the study.

### Exclusion criteria

Pregnant or lactating females, those with ECOG performance status > 2, impaired LV systolic function in which LVEF < 50%, documented coronary artery disease, significant valvular heart disease, history of cardiomyopathy or congestive heart failure (CHF), ALT > 3 times ULN, Patients already taking statins or any other lipid lowering drug or with a known hypersensitivity to any of the used drugs were excluded from the study.

### Patients’ selection and study design

All HER 2 positive breast cancer patients attending Clinical Oncology Department, Tanta University Hospital between October 2020 and October 2022 were tested for eligibility for our study, the total number was 60 patients, 7 patients were excluded (5 of them didn’t meet inclusion criteria and 2 patients refused participation), and 53 patients were recruited in the study, randomly divided into two groups based on days of admission: group 1 (control group) 26 patients didn’t receive rosuvastatin and group 2 (treatment group) 27 patients received 20 mg of oral rosuvastatin 24 h before the first cycle of chemotherapy and once daily for the rest of the follow-up period (6 months).

All patients received the same chemotherapy regimen: Doxorubicin 60 mg/m^2^ IV day 1 + Cyclophosphamide 600 mg/m^2^ IV day 1 cycled every 21 days for 4 cycles, followed by: Paclitaxel 80 mg/m^2^ by 1 h IV weekly for 12 weeks with Trastuzumab 4 mg/kg IV with first dose of paclitaxel followed by: Trastuzumab 2 mg/kg IV weekly.

**Fig. 1:**
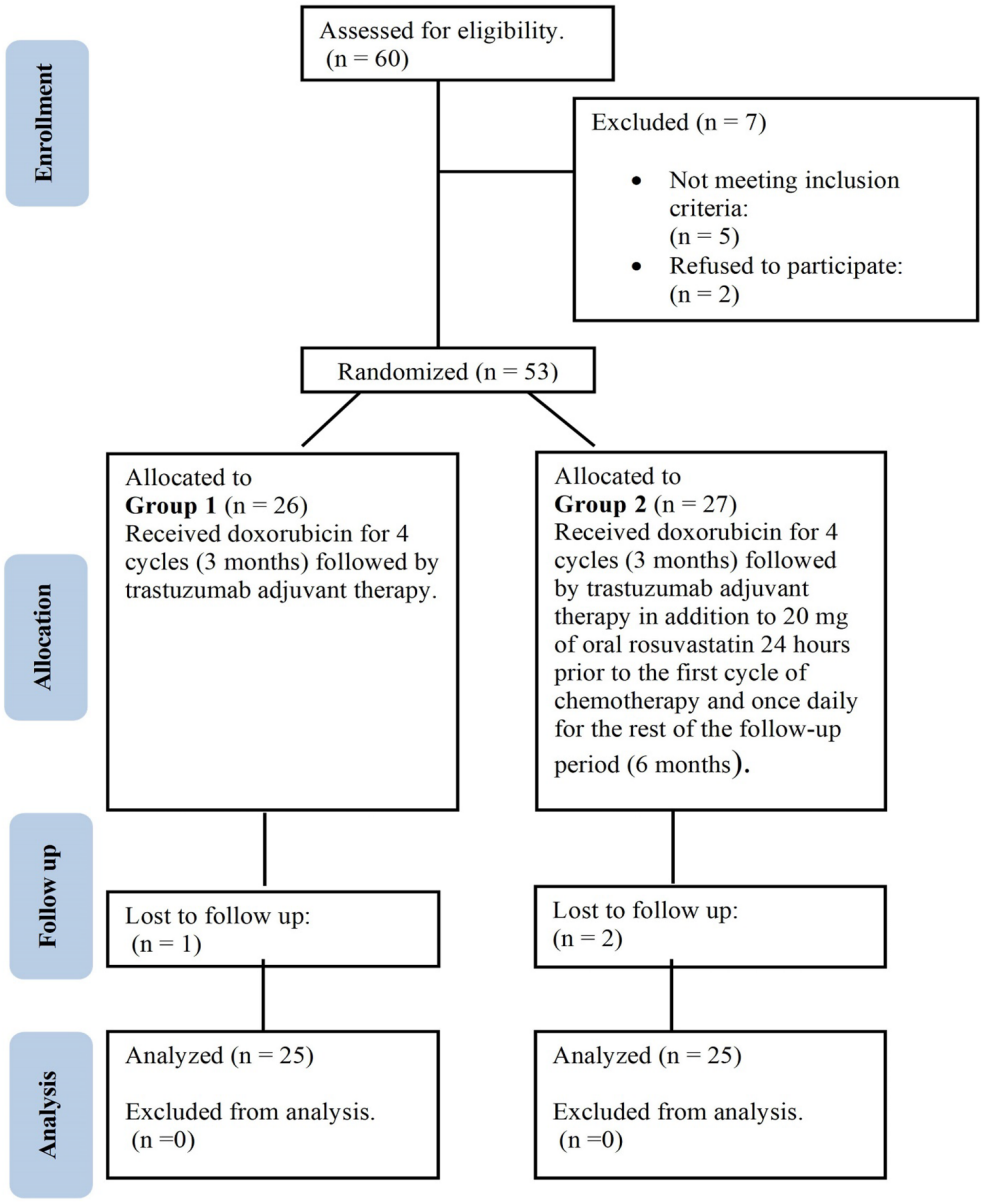
Consort flow diagram of the study

One patient was missed from follow-up in group 1 and two patients were missed in group 2, the analyzed number was 25patients in each group (Fig. 1). All the patients were subjected to complete history taking, clinical examination, weight and height measurement, body surface area (BSA) calculation. The chemotherapy dose was calculated according to patients’BSA. For all patients, ECHO was done to detect changes in LVEF, and blood samples were collected to evaluate serum levels of High sensitivity cardiac troponin I (hs-cTnI), Myeloperoxidase (MPO) and Interleukin-6 (IL-6). Serum level of Alanine aminotransferase (ALT) was also assessed for patients in both groups to detect any significant side effects of rosuvastatin on patients’liver function.

#### Echocardiography

Transthoracic echocardiography (Echo) was done for all patients 24 h before the initiation of drug therapy, after 3 months and after 6 months. Cardiotoxicity was defined as symptomatic heart failure or a decrease of left ventricular ejection fraction by ≥ 10% compared to the baseline value or to < 50% [6, 15].

#### Sample collection

For every patient, 5 ml of blood were drawn at plain tubes at the baseline (24 h before initiation of drug therapy), after 3 months and after 6 months. To extract serum, blood samples were allowed to clot, and then centrifuged. Each serum sample was divided to four portions to assess serum levels of hs-cTnI, MPO, IL-6 and ALT.

### Laboratory methods

Enzyme‑linked immunosorbent assay (ELISA) was used for measurement of serum levels of hs-cTnI, MPO, IL-6 using the technique of double-antibody sandwich ELISA and was conducted according to the manufacturer’s instructions (Shanghai SunRed Biological Technology Co., Ltd, China). Spectrophotometric detection was used to detect the serum concentrations of ALT procedures were done according to the manufacturer’s instructions (Agappe Diagnostics Ltd., Ernakulam, Kerala, India).

### Assessment of participants’adherence and side effects

Patients were regularly followed up through weekly calls and face-to-face meetings on three-weekly intervals at scheduled visits to evaluate their compliance with medication and to detect any reported side effects. The patients' adherence was assessed through counting the returned tablets and the rate of medication refills. Non-adherent patients were excluded from the study.

### Primary and secondary outcomes of the study

The primary outcomes were the change in LVEF after 3 months and 6 months of treatment compared to baseline and the detection of incidence of cardiotoxicity in both groups. The secondary outcome was the change in serum levels of hs-cTnI, MPO, IL-6 after 3 months and 6 months of treatment compared to baseline.

### Sample size calculation

G * Power 3.1 program was used to calculate the required sample size. Based on a previous study [16], the estimated sample size of 21 patients in each group would achieve a statistical power of 90% to detect the effect on the decline in LVEF (*α* error = 0.05, *β* error = 0.1). Assuming that the attrition rate is 15%, the sample size was 25 patients in each group.

### Statistical analysis

Statistical analysis was done using Statistical Package for Social Sciences (IBM SPSS Statistics version 26). The Shapiro–Wilk test was used to examine the normality of the data, since showed the data are normal. Numerical variables expressed by mean and standard deviation. Repeated measure ANOVA test was used to compare durations for each group individually. Independent t-test was used to compare between the two studied groups through each duration. Chi square test was used for categorical variables. Correlation analysis was used to show the relation between LVEF & (hs-TnI, IL-6 and MPO) and hs-TnI & (IL-6 and MPO). *P* value < 0.05(*) was considered significant difference & *P* value < 0.001(**) was considered highly significant difference.

## Results

### Demographic & clinical data of the studied patients

At baseline, No significant difference was detected between the two studied groups regarding age, BSA, side of breast cancer and history of diabetes mellitus, hypertension, and smoking. No significant difference was detected also in LVEF, serum levels of hs-cTnI, MPO, IL-6 and ALT. (Table 1).

**Table 1:** Demographic and clinical data of the studied groups

		Group 1 (control group)	Group 2 (treatment group)	*P* value
Age		47.92 ± 9.14	49.76 ± 9.51	0.489
BSA		1.71 ± 0.11	1.75 ± 0.08	0.248
LVEF (%)		68.96 ± 4.43	66.68 ± 4.81	0.534
Hs-cTnI (pg/ml)		205.83 ± 46.13	205.62 ± 52.12	0.998
MPO (ng/ml)		30.60 ± 4.96	32.50 ± 12.12	0.472
IL-6 (ng/L)		52.57 ± 17.20	62.09 ± 24.25	0.116
ALT (U/L)		19.84 ± 4.95	21.40 ± 6.01	0.322
Diabetes mellitus	Yes	6 (24%)	5 (20%)	0.733
	No	19 (76%)	20 (80%)	
Hypertension	Yes	6 (24%)	7 (28%)	0.747
	No	19 (76%)	18 (72%)	
Smoking	Yes	0 (0%)	0 (0%)	–
	No	25 (100%)	25 (100%)	
Side of breast cancer	Right	12 (48%)	11 (44%)	0.777
	Left	13 (52%)	14 (56%)	

Data expressed as mean ± standard deviation or number (%)

### Change in LVEF throughout the study period

**Fig. 2:**
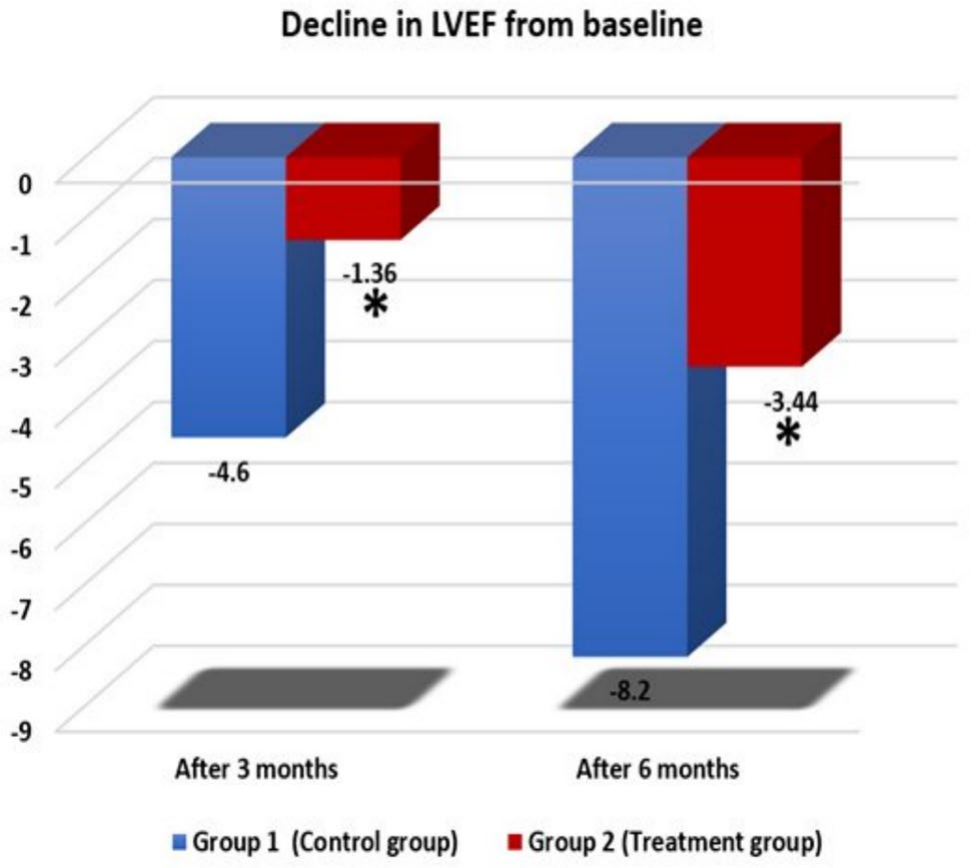
Decline in LVEF

There was no significant difference in LVEF between both groups at baseline. On the other hand, patients in rosuvastatin-treated group had a significantly lower decline in LVEF after 3 months and after 6 months compared to control group. (*P* = 0.036 and *P* = 0.002, respectively). (Fig. 2).

### Incidence of cardiotoxicity

A significant difference in incidence of cardiotoxicity was detected between the two studied groups (*P* = 0.037). Four patients in the control group experienced cardiotoxicity (16%) while no one was diagnosed with cardiotoxicity in rosuvastatin-treated group during the study period.

### Change in serum biomarkers’ levels throughout the study period

At baseline, there was no statistically significant difference between the two studied groups in serum levels of hs-cTnI, MPO, IL-6 and ALT.

Hs-cTnI was significantly lower in rosuvastatin-treated group compared to control group after 3 months (*P* = 0.026) and after 6 months (*P* = 0.046). There was a significant rise in hs-cTnI level in control group after 3 months and after 6 months compared to its baseline while rosuvastatin-treated group had non-significant change in hs-cTnI level after 3 months and after 6 months compared to its baseline.

Regarding MPO, no significant difference was observed between both groups after 3 months while patients in rosuvastatin-treated group had significantly lower MPO level after 6 months compared to patients in control group (*P* = 0.014). Compared to baseline values, control group had significantly higher MPO serum level after 3 months and after 6 months compared to its baseline while rosuvastatin-treated patients had no significant difference at any time point.

Compared to control group, rosuvastatin-treated group had significantly lower IL-6 after 3 months (*P* = 0.023) and after 6 months (*P* = 0.001). Compared to its baseline, control group had a highly significant rise in IL-6 after 3 months and after 6 months while rosuvastatin-treated group had a slight non-significant decrease in IL-6 at each time point.

ALT level was preserved in both groups throughout the study (Table 2).

**Table 2:** Serum biomarkers’ levels throughout the study period

	Group 1 (control group)	Group 2 (treatment group)	P value between the two studied groups
Hs-cTnI (pg/ml)			
At baseline	205.83 ± 46.13	205.62 ± 52.12	0.998
After 3 months	250.84 ± 89.09^#^	206.84 ± 57.40	0.026*
After 6 months	253.77 ± 84.98^#^	213.35 ± 49.69	0.046*
MPO (ng/ml)			
At baseline	30.60 ± 4.96	32.50 ± 12.12	0.472
After 3 months	35.58 ± 11.86^#^	29.81 ± 11.07	0.103
After 6 months	36.59 ± 11.78^#^	28.37 ± 10.94	0.014*
IL-6 (ng/L)			
At baseline	52.57 ± 17.20	62.09 ± 24.25	0.116
After 3 months	74.39 ± 20.90^##^	59.18 ± 24.92	0.023*
After 6 months	73.56 ± 18.23^##^	51.17 ± 19.40	0.001**
ALT (U/L)			
At baseline	19.84 ± 4.95	21.40 ± 6.01	0.322
After 3 months	20.03 ± 5.82	22.84 ± 4.46	0.062
After 6 months	21.60 ± 6.24	25.79 ± 9.74	0.115

Data expressed as mean ± standard deviation

*Indicates statistically significant difference between the two studied groups at *P* < 0.05 for independent *t*-test

^#^Indicates statistically significant difference within the same group compared to baseline values at *P* < 0.05 for Repeated measure ANOVA test

### Correlations between LVEF and serum biomarkers

At the end of the study, there was a highly significant strong negative relationship between LVEF and both hs-cTnI &MPO in both groups. A highly significant strong negative relationship was found between LVEF and IL-6 in control group but a non-significant weak negative relationship in rosuvastatin-treated group. A highly significant strong positive correlation was found between Hs-cTnI and both MPO & IL-6 in both groups (Table 3).

**Table 3:** Correlation between LVEF and serum biomarkers

Pearson correlation (r) after 6 months of treatment					
		LVEF		Hs-cTnI	
		*r*	*P* value	*r*	*P* value
Hs-cTnI	Group 1 (control group)	− 0.844	< 0.001**	–	–
	Group 2 (treatment group)	− 0.979	< 0.001**	–	–
MPO	Group 1 (control group)	− 0.939	< 0.001**	0.959	< 0.001**
	Group 2 (treatment group)	− 0.948	< 0.001**	0.649	< 0.001**
IL-6	Group 1 (control group)	− 0.920	< 0.001**	0.977	< 0.001**
	Group 2 (treatment group)	− 0.368	0.064	0.988	< 0.001**

*Indicates statistical significance at *P* < 0.05

### Reported side effects

Patients in the two groups reported the same side effects: nausea, abdominal pain, myalgia, headache, asthenia, and dizziness. No significant difference was found between both groups.

## Discussion

The majority of HER2 positive breast cancer patients are treated with anthracycline-based chemotherapy followed by anti HER2 therapy (mainly trastuzumab) to enhance response to treatment [17].Even when treatments are given sequentially, cardiotoxicity when trastuzumab is coupled with an anthracycline still warrants careful attention [18]. Clinical trials showed that administration of trastuzumab following anthracycline-based chemotherapy was associated with higher incidence of cardiotoxicity [19]. Cardiac-protective treatments that decrease myocardial damage may help to prevent chemotherapy-induced cardiotoxicity [20].

Statins have many pleotropic effects besides their ability to decrease low density lipoprotein cholesterol [21]. Rosuvastatin has antioxidant and anti-inflammatory effects that prevent DNA damage and may provide cardioprotective effect [13].Therefore, we examined the cardioprotective effect of rosuvastatin in chemotherapy-induced cardiotoxicity in HER2 positive breast cancer patients.

LVEF is a frequently used echocardiographic parameter for evaluating cardiac function during chemotherapy [21].We defined cardiotoxicity as symptomatic heart failure or a decrease of left ventricular ejection fraction by ≥ 10% compared to the baseline value or to < 50% [6, 15]. Based on that definition, In our study, rosuvastatin-treated group had a significant lower decline in LVEF after 3 months and after 6 months compared to control group.

Our results agrees with Nabati et al. who investigated the cardioprotective effect of rosuvastatin (20 mg/day) on female breast cancer patients on anthracycline-based chemotherapy and found that LVEF of the intervention group was not statistically altered while the LVEF of the placebo group showed a significant drop [21].

Our finding about LVEF is also going with Stone et al. and Acar et al. who reported that patients received statins had significantly lower decline in LVEF than the control group in cancer patients received anthracycline-based chemotherapy for different cancers [16, 22].

In our study, 16% of patients in control group experienced cardiotoxicity while no one in rosuvastatin-treated group during the study period, which is going with other previous studies that observed that number of patients who had LVEF < 50% in patients received statins was lower than control [18, 20].

In cardio-oncology, change in serum biomarkers is more sensitive than drop in LVEF so we can assess levels of serum biomarkers to detect subclinical cardiotoxicity [11, 23].Studies conducted on patients undergrowing high-dose chemotherapy ascertained that there is strong relationship between troponin I (TnI) rise and LVEF decline so we can consider TnI as an early-predictor of myocardial damage [24, 25].Investigators also noted that TnI positive predictive value was 84%, and its negative predictive value was 99% [24].

In the present study, control group had a significant rise in hs-cTnI after 3 months and 6 months compared to its baseline, also a strong significant negative correlation was detected between LVEF and hs-cTnI in both groups, which agrees with several previous studies that reported that serum levels of TnI elevated in breast cancer patients underwent sequential therapy with doxorubicin and trastuzumab and were associated with increased risk for significant LVEF drop [11, 26, 27].

In our study, Hs-cTnI was significantly lower in rosuvastatin-treated group compared to control group after 3 months and 6 months which is in accordance with other previous preclinical studies that reported that rosuvastatin resulted in significant reduction in TnI compared to control in doxorubicin-induced cardiotoxicity and trastuzumab-induced cardiotoxicity [28–30].

This can be explained by the ability of rosuvastatin to decrease oxidative stress in cardiomyocytes via its antioxidant effect through reducing the production of ROS and upregulation of antioxidant enzymatic defense mechanism and also its ability to suppress cardiomyocytes apoptosis [30–33].

MPO is a marker of oxidative stress and inflammation that is elevated in patients with heart failure and cardiac dysfunction, it is known to be associated with disease progression and severity [23, 34, 35].MPO is linked to inflammatory process through its enzymatic activity and generated oxidants that lead to tissue damage [36]. It also plays an important role in doxorubicin-induced cardiotoxicity by promoting oxidation of sarcomeric proteins and accelerating cardiac inflammation as well as apoptosis through p38 mitogen-activated protein kinase (MAPK) signalling, and MPO genetic ablation or drug-based inhibition of MPO can protect from cardiac dysfunction [37].

In our study we found that control group had significantly higher MPO serum level after 3 months and after 6 months compared to its baseline, which agrees with Januzzi et al. who found that serum levels of MPO were significantly elevated after 3 months and early changes in MPO were associated with subsequent cardiotoxicity in patients with breast cancer underwent sequential therapy with doxorubicin and trastuzumab [11].

In addition, our rosuvastatin-treated patients had significantly lower MPO level after 6 months compared to control group, and there was non-significant change in MPO level after 3 and 6 months compared to their baseline which is going with Andreou et al. results that reported that a short term treatment with low-dose rosuvastatin treatment significantly lowered MPO in heart failure patients [35]. This may be explained by the ability of satins to suppress MPO gene expression which contributes to their pleiotropic effects [38].

In the present study, we also observed that MPO had a strong correlation with LVEF and Hs-cTnI in both groups which reflects the role of MPO in chemotherapy-induced cardiotoxicity.

IL-6 is a biomarker for inflammation linked to higher risk of cardiovascular events [23].CHF patients have higher levels of circulating IL-6.The degree of left ventricular dysfunction is correlated with IL-6 levels, which are also highly predictive of subsequent clinical outcomes [39].Doxorubicin upregulates the expressions of pro-inflammatory cytokines, including IL-6, through activation of the Nuclear factor kappa B (NF-κB), triggering the progression of adverse cardiac events [40]. IL-6 exacerbates oxidative stress-induced mitochondrial dysfunction and leads to cardiomyocyte apoptosis. In addition, IL-6 alters calcium handling and decreases cardiac contractility, which causes diastolic dysfunction and arrhythmia [41].

In our study, patients in control group showed significant increase in IL-6 level after 3 months and after 6 months compared to its baseline, which agrees with preclinical studies that reported that doxorubicin-induced cardiotoxicity in rats was associated with significant increase in IL-6 [10].It is also going with an animal study that found an increase in IL-6 gene expression in cardiac tissue of trastuzumab-induced cardiotoxicity rats [42].

In the present study, we also observed that IL-6 had a significant correlation with LVEF and Hs-cTnI which may express the role of IL-6 in chemotherapy-induced cardiotoxicity.

On other hand, our rosuvastatin-treated group showed non-significant change in IL-6 level after 3 and 6 months compared to baseline. In addition, IL-6 level in rosuvastatin-treated patients was significantly lower than their respective value of patients in control group at each time point, which is going with other previous clinical studies that found that rosuvastatin significantly decreased IL-6 in patients with ST-segment elevated myocardial infarction, hypertension and dyslipidaemia [43, 44].Our results also agrees with a preclinical study found that rosuvastatin significantly decreased IL-6 in trastuzumab-induced cardiotoxicity mice [30].The decrease in IL-6 may be explained by ability of rosuvastatin to inhibit activation of the Nuclear factor kappa B (NF-κB) [45].

Regarding safety of rosuvastatin, no added side effects were reported in both groups throughout the study. ALT wasn’t altered in the both groups which means that rosuvastatin was well-tolerated with no added side effects.

Our results revealed that rosuvastatin can protect against cardiotoxicity associated with breast cancer therapy especially doxorubicin and trastuzumab documented by LVEF and proved by specific cardiac inflammatory marker, hs-cTnI and non-specific markers, MPO and IL-6.

### Limitations

Our study was single -centered, short follow-up duration, limited number of patients, no placebo treatment was used, other ECHO parameters as left ventricular wall thickness and diastolic parameters may have been studied, strain measurement wasn’t used to assess cardiac function, effect of rosuvastatin on cancer treatment or other chemotherapy may have been studied.

## Conclusion

Our work revealed that rosuvastatin is a promising agent to protect against chemotherapy-induced cardiotoxicity.

## Data Availability

All data and materials are available upon request.
